# A Scoping Review of Transdermal Buprenorphine Use for Non-surgical Pain in the Pediatric Population

**DOI:** 10.7759/cureus.5954

**Published:** 2019-10-21

**Authors:** Thomas S Haupt, Michael Smyth, Marie-Claude Gregoire

**Affiliations:** 1 Palliative Care Pediatrics, Dalhousie Medical School, Halifax, CAN

**Keywords:** palliative, pediatrics, buprenorphine transdermal patch, safety, pain

## Abstract

A preliminary evaluation to review the scope and quality of evidence surrounding transdermal buprenorphine use in the pediatric setting for non-surgical pain was conducted. Our review revealed limited data available on the use of transdermal buprenorphine in pediatric patients. Most studies surrounding this subject involve accidental ingestion of buprenorphine and its use in the treatment of neonatal abstinence syndrome. While indicated for use only in adult populations, small studies have shown encouraging results in reducing pain in children with few, if any, adverse effects. This is reassuring from a clinical perspective, as we hope to highlight the available evidence and invite researchers to expand future studies. Through this review, we have identified significant gaps in the literature surrounding the safety and use of buprenorphine in the pediatric population. To our knowledge, there are no major studies investigating this subject, and it is our hope that future studies will explore the use of transdermal buprenorphine as an alternative pain management technique in pediatrics. The intent of our scoping review is to highlight the lack of research in this area; therefore, future studies may be conducted to support its use in North America.

## Introduction and background

Buprenorphine is an opioid medication commonly used in the treatment of severe surgical and cancer pain, acting as a partial µ-opioid receptor agonist and a κ-receptor antagonist in the central nervous system [1]. Though an effective medication in the adult population, it is not frequently used in the pediatric population. This may be due, in part, to the fact that in Canada and the United States, buprenorphine is not indicated for use in the pediatric community. Literature surrounding the use of buprenorphine in pediatrics is sparse, but it is primarily limited to case reports. Despite not being indicated for use in pediatrics, transdermal buprenorphine has been used off-label for the treatment of severe non-surgical and cancer pain in the pediatric setting for many years. Transdermal use in pediatrics is preferred because buprenorphine reaches steady state plasma levels over seven days in this modality and avoids the need of intravenous opioid use, which can be difficult to manage as an outpatient [2]. Ruggiero et al. found that transdermal buprenorphine used in the setting of pediatric cancer pain led to a significant improvement in several aspects of quality of life, including sleeping and level of activity [3,4]. Importantly, studies investigating transdermal buprenorphine use in pediatrics do not report any major adverse drug effects (ADE) to the medication, with only expected mild negative reactions (nausea, headache, constipation, and patch site pruritus and erythema) typically reported [3]. Given the scarce literature surrounding pediatric buprenorphine use, this scoping review aims to consolidate the literature surrounding transdermal buprenorphine use in a pediatric setting, most importantly any evidence regarding drug safety. It is our hope that this review will highlight the safety of pediatric buprenorphine use from the current literature and the need for further research around this topic.

## Review

Methods

In this scoping review, we screened PubMed, Embase and Cumulative Index to Nursing and Allied Health Literature (CINAHL) databases. Databases were combined using Medical Subject Headings (MeSH) terms adapted from Leclercq et al. (Table 1) [5]. All studies, including case reports and studies with varying routes of buprenorphine administration, containing a patient population of n≥1 were included, given the scarcity of literature on the topic. Exclusion criteria included any patient above the age of 18 years, buprenorphine use in regard to addiction, accidental use, research limited to letters and commentaries, posters, abstract only, protocols, caudal, epidural and spinal anesthesia.

In order to properly organize our database results, the Preferred Reporting Items for Systematic Reviews and Meta -Analyses (PRISMA) was implemented. PRISMA is an evidence-based guideline to improve the reporting of systematic and scoping reviews [6]. Covidence software (Covidence, Melbourne, Australia) was used to screen and filter available literature, with each primary author screening results independently. Screening discrepancies were resolved by the third author (Gregoire, MC). Once studies were screened and deemed appropriate for review, data extraction was completed on each paper by each author independently. This was done using a data extraction form developed by the authors. Data were then compiled and reviewed. 

**Table 1:** Databases and MeSH terms used for study retrieval

Database	MeSH Search Terms
PubMed	((((((("Buprenorphine"[Mesh] OR “anorfin”[tiab] OR “belbuca”[tiab] OR “buprenex”[tiab] OR “buprenorphine”[tiab] OR “buprenorphine hydrochloride”[tiab] OR “buprex”[tiab] OR “buprine”[tiab] OR “butrans”[tiab] OR “cl 112, 302”[tiab] OR “cl 112302”[tiab] OR “cl112, 302”[tiab] OR “cl112302”[tiab] OR “finibron”[tiab] OR “lepetan”[tiab] OR “nih 8805”[tiab] OR “nih8805”[tiab] OR “norphin”[tiab] OR “pentorel”[tiab] OR “prefin”[tiab] OR “probuphine”[tiab] OR “rx 6029 m”[tiab] OR “rx 6029m”[tiab] OR “rx6029m”[tiab] OR “somnena”[tiab] OR “sublocade”[tiab] OR “subutex”[tiab] OR “temgesic”[tiab] OR “um952”[tiab] OR “um 952”[tiab] OR “transtec”[tiab]))) AND (Infan* OR newborn* OR new-born* OR perinat* OR neonat* OR baby OR babies OR toddler* OR minors OR minors* OR boy OR boys OR boyfriend OR boyhood OR girl* OR kid OR kids OR child OR childhood OR children OR schoolchild* OR schoolchild OR school child[tiab] OR school child*[tiab] OR adolescen* OR juvenil* OR youth* OR teen* OR under*age* OR pubescen* OR pediatrics[mh] OR pediatric* OR paediatric* OR peadiatric* OR school [tiab] OR school*[tiab] OR prematur* OR preterm*)))))
Embase	'buprenorphine'/exp OR 'anorfin':ti,ab OR 'belbuca':ti,ab OR 'buprenex':ti,ab OR 'buprenorphine':ti,ab OR 'buprenorphine hydrochloride':ti,ab OR 'buprex':ti,ab OR 'buprine':ti,ab OR 'butrans':ti,ab OR 'cl 112, 302':ti,ab OR 'cl 112302':ti,ab OR 'cl112, 302':ti,ab OR 'cl112302':ti,ab OR 'finibron':ti,ab OR 'lepetan':ti,ab OR 'nih 8805':ti,ab OR 'nih8805':ti,ab OR 'norphin':ti,ab OR 'pentorel':ti,ab OR 'prefin':ti,ab OR 'probuphine':ti,ab OR 'rx 6029 m':ti,ab OR 'rx 6029m':ti,ab OR 'rx6029m':ti,ab OR 'somnena':ti,ab OR 'sublocade':ti,ab OR 'subutex':ti,ab OR 'temgesic':ti,ab OR 'um952':ti,ab OR 'um 952':ti,ab OR 'transtec':ti,ab
CINAHL	(((((((MH "Buprenorphine" OR TI “anorfin” OR AB “anorfin” OR TI “belbuca” OR AB “belbuca” OR TI “buprenex” OR AB “buprenex” OR TI “buprenorphine” OR AB “buprenorphine”OR TI “buprenorphine hydrochloride” OR AB “buprenorphine hydrochloride” OR TI “buprex” OR AB “buprex” OR TI “buprine” OR AB “buprine” OR TI “butrans” OR AB “butrans” OR TI “cl 112, 302” OR AB “cl 112, 302” OR TI “cl 112302” OR AB “cl 112302” OR TI “cl112, 302” OR AB “cl112, 302” OR TI “cl112302” OR AB “cl112302” OR TI “finibron” OR AB “finibron” OR TI “lepetan” OR AB “lepetan” OR TI “nih 8805” OR AB “nih 8805” OR TI “nih8805” OR AB “nih8805” OR TI “norphin” OR AB “norphin” OR TI “pentorel” OR AB “pentorel” OR TI “prefin” OR AB “prefin” OR TI “probuphine” OR AB “probuphine” OR TI “rx 6029 m” OR AB “rx 6029 m” OR TI “rx 6029m” OR AB “rx 6029m” OR TI “rx6029m” OR AB “rx6029m” OR TI “somnena” OR AB “somnena” OR TI “sublocade” OR AB “sublocade” OR TI “subutex” OR AB “subutex” OR TI “temgesic” OR AB “temgesic” OR TI “um952” OR AB “um952” OR TI “um 952” OR AB “um 952” OR TI “transtec” OR AB “transtec”))) AND (Infan* OR newborn* OR new-born* OR perinat* OR neonat* OR baby OR babies OR toddler* OR minors OR minors* OR boy OR boys OR boyfriend OR boyhood OR girl* OR kid OR kids OR child OR childhood OR children OR schoolchild* OR schoolchild OR TI school child OR AB school child OR TI school child* OR AB school child* OR adolescen* OR juvenil* OR youth* OR teen* OR under*age* OR pubescen* OR MH pediatrics OR pediatric* OR paediatric* OR peadiatric* OR TI school OR AB school OR TI school* OR AB school* OR prematur* OR preterm*)))))

Results

**Figure 1:**
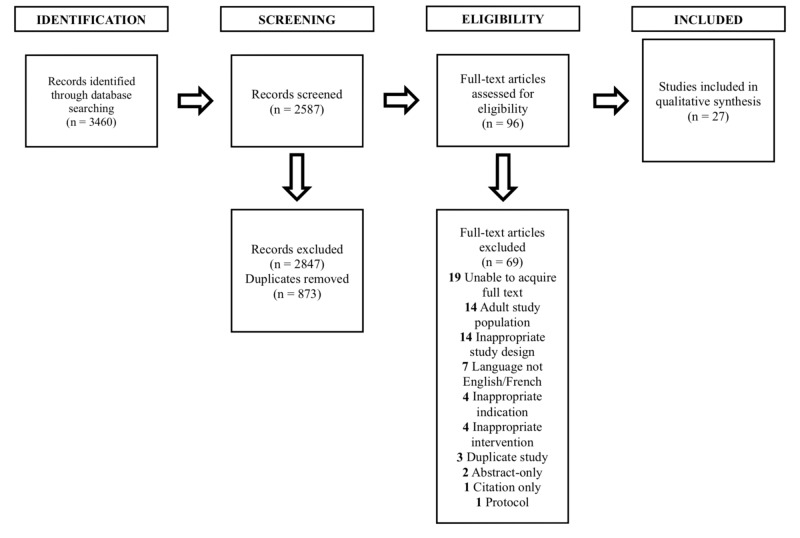
PRISMA flowchart for the development of the reference dataset.

Our search through PubMed, Embase and CINAHL revealed 2587 studies. Of the 2587 studies screened for transdermal buprenorphine use in the pediatric population, only 96 studies were eligible for further analysis based on our inclusion and exclusion criteria. Of the 96 studies analyzed, 27 went on for further analysis following our exclusion criteria (Figure 1). Most of these studies were found to be either commentaries or systematic reviews (Figure 2). In the literature, the common ADE aforementioned were found in pediatric patients using transdermal buprenorphine, the most common being nausea, vomiting and local skin reactions around transdermal system sites (Figure 3). The majority (63%) of studies were published in 2010 or later (Figure 4), with 28% of the studies utilizing a transdermal formulation of buprenorphine (Figure 5).

Discussion

During this scoping review, it was found that the majority (N=2487, 96.1%) of the literature surrounding buprenorphine use in pediatrics fits under the category of improper use (accidental use, addiction and neonatal abstinence syndrome). Improper use of buprenorphine was excluded from our study. Furthermore, much of the clinical research regarding pediatric buprenorphine use comes in the form of commentaries, reviews and case reports with only one trial discovered, as done by Ruggiero et al. in 2013 [4]. In addition, 63% were published in, or after, the year 2010. We believe this is due to the advent of the Butrans transdermal buprenorphine system in 2010, which allows studies investigating buprenorphine use in pediatrics to become more feasible [7]. The transdermal formulation may provide a more promising treatment modality in pediatrics, as oral and intravenous routes can prove difficult in this population [2,8,9].

The use of any opioid carries a risk of the common ADE including, but not limited to, nausea and vomiting, delirium, constipation, pruritus, sedation and respiratory depression [10]. Our scoping review was devoid of any adverse drug reactions outside of the ADE mentioned above, with the most common being mild reactions such as nausea/vomiting, headaches, constipation, and transdermal site pruritus and erythema, the latter of which resolved quickly when the patch was removed [3,4,9]. Furthermore, as mentioned previously, Ruggiero et al. were able to demonstrate that pediatric transdermal buprenorphine use could significantly improve a patient’s quality of life for 11 (69%) of the 16 children enrolled in that study [4]. Transdermal buprenorphine has been shown to adequately control palliative cancer pain in adults and improve the quality of life in patients with palliative cancer pain [11]. ADE seem to be reduced with transdermal opioids like buprenorphine because of the avoidance of first-pass metabolism, which augments the bioavailability and therefore allows physicians to prescribe lower doses [12]. Thus, with transdermal buprenorphine we can get better pain control with lower doses and fewer ADE. Transdermal buprenorphine is preferred by patients because it is non-invasive, simple to use, does not disrupt one’s activities of daily living and are tolerated by patient who cannott swallow or have low tolerance for more traditional opioids like morphine [13]. The transdermal patch has obvious advantages in children who have difficulty swallowing and tolerating intravenous catheters. In adults, pain control and symptom management are correlated with survival and increased quality of life [14]. It is our hope that these same benefits seen in adults will also be seen in children.

Despite much of the literature coming in the form of case reports and commentaries, the prevailing theme amongst the literature is that buprenorphine has the same expected ADE in children as it does in adults [4,15].

## Conclusions

Currently, the transdermal form of buprenorphine is primarily used off-label in pediatrics and as such, it can be both difficult and expensive for patients and families to obtain through insurance plans or drug formularies. The goal of this scoping review was to provide the most up-to-date information regarding the safety of buprenorphine use in the pediatric population. Given the absence of major adverse drug reactions, transdermal buprenorphine may provide a safe, convenient alternative to current pain treatment therapies in pediatrics. We hope that this review can encourage future research focused at effectively demonstrating the safety of buprenorphine in pediatrics. This future research, if it were to be done, could target children who are opioid naïve to traditional opioids, cannot swallow or their pain is not being adequately controlled. 

As it has such potential, and once safety is properly established, buprenorphine could become an important treatment option in the management of pediatric pain.

## References

[1] Vadivelu N, Anwar M (2010). Buprenorphine in postoperative pain management. Anesthesiol Clin.

[2] Kapil RP, Cipriano A, Friedman K (2013). Once-weekly transdermal buprenorphine application results in sustained and consistent steady-state plasma levels. J Pain Symptom Manage.

[3] Attina G, Ruggiero A, Maurizi P (2009). Transdermal buprenorphine in children with cancer-related pain. Pediatr Blood Cancer.

[4] Ruggiero A, Coccia P, Arena R (2013). Efficacy and safety of transdermal buprenorphine in the management of children with cancer-related pain. Pediatr Blood Cancer.

[5] Leclercq E, Leeflang MM, van Dalen EC (2013). Validation of search filters for identifying pediatric studies in PubMed. J Pediatr.

[6] Moher D, Liberati A, Tetzlaff J (2009). Preferred reporting items for systematic reviews and meta-analyses: the PRISMA statement. PLoS Med.

[7] Pastore MN, Kalia YN, Horstmann M (2015). Transdermal patches: History, development and pharmacology. Br J Pharmacol.

[8] Delgado-Charro MB, Guy RH (2014). Effective use of transdermal drug delivery in children. Adv Drug Deliv Rev.

[9] Michel E, Anderson BJ, Zernikow B (2011). Buprenorphine TTS for children--a review of the drug's clinical pharmacology. Paediatr Anaesth.

[10] Ripamonti CI, Santini D, Maranzano E (2012). Management of cancer pain: ESMO Clinical Practice Guidelines. Ann Oncol.

[11] Clement PM, Beuselinck B, Mertens PG (2013). Pain management in palliative cancer patients: a prospective observational study on the use of high dosages of transdermal buprenorphine. Acta Clinica Belgica.

[12] Vithlani RH, Baranidharan G (2010). Transdermal opioids for cancer pain management. Rev Pain.

[13] Skaer TLJ (2014). Dosing considerations with transdermal formulations of fentanyl and buprenorphine for the treatment of cancer pain. J Pain Res.

[14] Temel JS, Greer JA, Muzikansky A (2010). Early palliative care for patients with metastatic non-small-cell lung cancer. New Engl J Med.

[15] Tassinari D, Sartori S, Tamburini E (2008). Adverse effects of transdermal opiates treating moderate-severe cancer pain in comparison to long-acting morphine: a meta-analysis and systematic review of the literature. J Palliat Med.

